# Bridging MBD3 functions in developmental biology and glioma heterogeneity to immune remodeling with future perspectives

**DOI:** 10.3389/fimmu.2026.1830872

**Published:** 2026-06-18

**Authors:** Nhu Thi Quynh Mai, Byoung-San Moon

**Affiliations:** Department of Medical Biotechnology, Yeungnam University, Gyeongsan, Republic of Korea

**Keywords:** epigenetics regulation, glioblastoma, MBD3, microglia, neoplastic stem cells, tumor escape and relapse, tumor microenvironment

## Abstract

Glioblastoma (GBM) remains one of the most lethal and treatment-refractory brain tumors, characterized by rapid recurrence and profound resistance to conventional therapies. The mechanisms underlying GBM resilience converge on two interdependent axes: intratumoral heterogeneity and adaptive tumor microenvironment (TME) remodeling, which engage in reciprocal crosstalk to promote immune evasion, invasion, and tumor persistence. Epigenetic regulation has emerged as a central determinant of tumor plasticity and cellular adaptability within this context. Methyl-CpG-binding domain protein 3 (MBD3), a core structural and regulatory component of the NuRD complex, represents a functionally distinctive epigenetic regulator that operates beyond canonical methylation-dependent pathways. With well-established roles in lineage commitment and neural development, MBD3 has been increasingly implicated in GBM progression, glioma stem cell maintenance, and therapeutic resistance. In parallel, GBM actively co-opts non-neoplastic TME components - including microglia, the resident innate immune sentinels of the central nervous system - which exert context-dependent tumor-supportive and tumor-suppressive functions. Understanding how MBD3 shapes tumor–immune interactions and modulates innate immune regulation is therefore of growing biological and translational importance. This review provides an overview of the MBD protein family and delineates the multifaceted roles of MBD3 in developmental and GBM biology, with particular emphasis on tumor-intrinsic heterogeneity and TME remodeling. By integrating epigenetic, cellular, and immunological perspectives, we highlight emerging evidence linking MBD3 to tumor plasticity and immune modulation, and propose hypothetical regulatory frameworks that warrant experimental investigation. Clarifying the context-dependent functions of MBD3 may yield mechanistic insights into GBM pathogenesis and inform the development of novel epigenetic therapeutic strategies.

## 1 Introduction

Glioblastoma (GBM), classified by the World Health Organization (WHO) as grade 4 central nervous system tumor, is the most common and aggressive primary brain malignancy in adults and is characterized by rapid proliferation and diffuse infiltration (1). Current standard treatment involves maximal surgical resection followed by adjuvant chemotherapy (2–4) and radiotherapy (5). Complementary therapeutic strategies, such as tumor-treating fields (6, 7) and immunotherapy-based approaches (8–10), are increasingly being integrated into clinical practice. Nevertheless, patient prognosis remains poor, with median survival of approximately 15 months and long-term survival exceedingly rare (11). The persistent therapeutic failure of GBM is largely driven by its profound cellular, metabolic, and microenvironmental heterogeneity, which promotes adaptive resistance and tumor recurrence.

Among the major mechanisms underlying GBM invasion and recurrence, epigenetic dysregulation has emerged as a central contributor. Unlike static genetic alterations, epigenetic modifications, encompassing DNA methylation, histone and chromatin remodeling, and non-coding RNA-mediated regulation, are highly dynamic, rendering them readily responsive to environmental stimuli. These inherently reversible regulatory mechanisms enable coordinated modulation of multiple transcriptional programs and signaling pathways, thereby facilitating tumor cell plasticity. Such plasticity allows GBM cells to adopt therapy-tolerant states under microenvironmental or treatment-induced stress, repopulate following therapeutic pressure, and subsequently re-establish malignancy (12–15).

Methyl-CpG binding domain protein 3 (MBD3) has emerged as an important epigenetic regulator in gliomagenesis. As a core component of the nucleosome remodeling and deacetylase (NuRD) complex, MBD3 is essential for maintaining glioma stem-like cell (GSC) identity and is preferentially expressed in aggressive tumor states both *in vitro* and *in vivo* (16–18). In addition, MBD3 has been implicated in modulating tumor sensitivity to alkylating chemotherapeutic agents (18) and in regulating immune evasion pathways (17). Collectively, these findings, together with its well-established roles in lineage specification and developmental programming (19–22), position MBD3 as a putative epigenetic integrator, linking chromatin remodeling to cellular plasticity and tumor progression in GBM.

Despite growing recognition of its biological importance, many mechanistic aspects of MBD3 function in GBM remain incompletely understood. Emerging evidence suggests that MBD3 influences diverse regulatory processes, encompassing tumor-intrinsic transcriptional programs, non-coding RNA networks, chromatin architecture, and tumor microenvironment (TME) interactions. However, the pathways coordinating these functions remain poorly defined. A deeper mechanistic understanding of MBD3-mediated regulation, particularly in the context of tumor heterogeneity, adaptive phenotypic transitions, and microenvironmental crosstalk, may yield critical insights into the molecular drivers of GBM progression and therapeutic resistance.

Within the GBM TME, microglia represent a particularly important yet relatively understudied cellular compartment. These resident immune cells exhibit dual anti-tumor and pro-tumor functions and can transition between functional states in response to contextual cues (23). Given the emerging role of MBD3 in immune regulation, determining whether MBD3 contributes to microglial reprogramming, either through cell intrinsic regulation or indirectly via tumor-derived signaling, may reveal additional mechanisms underlying immune evasion in GBM.

This review summarizes the multifaceted roles of MBD3 in GBM biology, with particular emphasis on its contributions to tumor heterogeneity, cellular plasticity, and TME regulation. Building on current evidence, we further propose putative mechanisms through which MBD3 may influence microglial polarization and immune modulation in GBM. By integrating current insights across chromatin regulation, developmental programming, and TME dynamics, this review highlights the emergence significance of MBD3 as a potential mechanistic and therapeutic target in GBM.

## 2 MBD3 in chromatin regulation and epigenetic plasticity

MBD3 serves as a key regulator of chromatin organization and epigenetic plasticity. As a core structural component of the NuRD complex, MBD3 stabilizes complex architecture through coiled-coil domain-mediated inter-subunit interactions, coordinating chromatin remodeling with histone deacetylation (24). Unlike canonical MBD family members that preferentially bind methylated CpG DNA (**Box 1**), MBD3 exhibits reduced affinity for 5-methylcytosine (5mC) and instead preferentially associates with 5-hydroxymethylcytosine (5hmC)-enriched chromatin regions (34). Given that 5hmC is typically associated with transcriptionally active or permissive chromatin states, this property positions MBD3 as an important intermediary linking TET-mediated DNA demethylation to NuRD-dependent chromatin remodeling (35). Through association with the NuRD complex, MBD3 contributes to the establishment of repressed or poised chromatin states at 5hmC-enriched loci, enabling context-dependent regulation of transcriptional programs involved in development and lineage specification (**Figure 2A**).

Box 1 **The MBD Protein Family: Structural and Functional Diversity**

The methyl-CpG-binding domain (MBD) protein family comprises a group of epigenetic regulators that interpret DNA methylation signals and modulate chromatin states to regulate gene expression. Members of this family are characterized by a conserved methyl-CpG-binding domain, which adopts a wedge-shaped structure composed of a β-sheet positioned over an α-helix and flanking loop regions. Amino acid residues within two β-strands and the N-terminal region adjacent to the α-helix contact methylated cytosine residues within the major groove of DNA, enabling selective recognition of methylated cytosine-phosphate-guanine (CpG) dinucleotides (25). CpG sites, particularly those enriched within gene promoter regions, represent major hotspots for DNA methylation and play central roles in transcriptional regulation (26).

**Figure 1:**
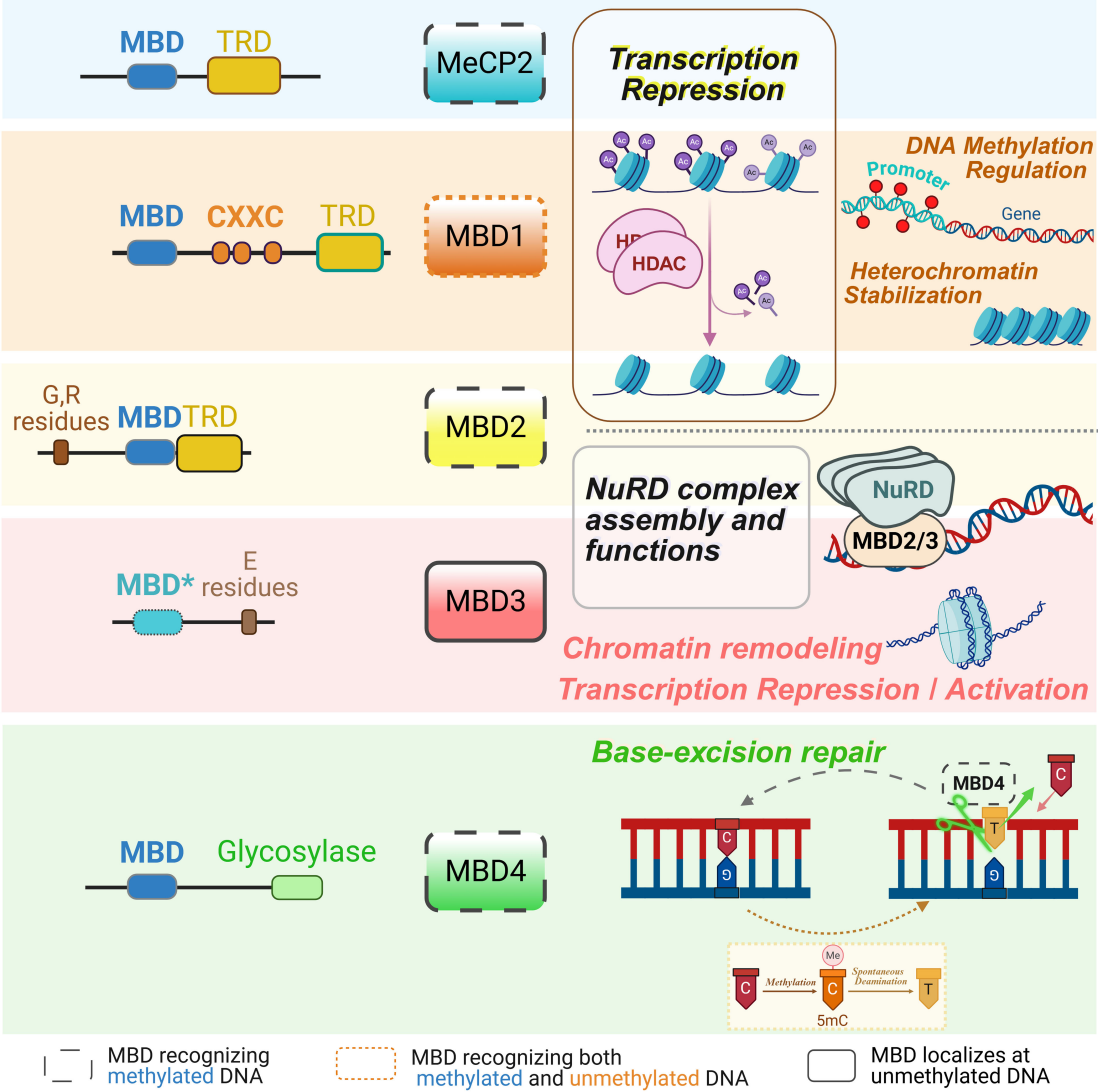
Domain organization and functional specialization of MBD family proteins. Schematic comparison of the domain architecture and key functional properties of MeCP2, MBD1, MBD2, MBD3, and MBD4. MBD, methyl-CpG-binding domain; TRD, transcriptional repression domain; CXXC, CXXC-type zinc finger domain; G, glycine; R, arginine; E, glutamate. MeCP2 , MBD2 and MBD2 mediate transcriptional repression through recruitment of HDAC-containing complexes. MBD1 also contributes to DNA methylation maintenance and heterochromatin stabilization. MBD2 and MBD3 are involved in NuRD complex assembly and function; MBD3 specifically enables chromatin remodeling and context-dependent transcriptional repression or activation. MBD4 functions in base-excision repair by recognizing and excising thymine mismatched with guanine at 5mC deamination sites. Domain boundaries are not to scale. Created with BioRender.com.

To date, five principal members of the MBD protein family—MeCP2, MBD1, MBD2, MBD3, and MBD4—have been extensively characterized. Although these proteins share the conserved MBD domain, they exhibit substantial functional diversity arising from distinct auxiliary domains that confer specialized regulatory properties (**Figure 1**).

MeCP2, MBD1, and MBD2 each harbor transcriptional repression domains (TRDs), although these are structurally non-homologous across family members. Through these regions, the proteins recruit co-repressor complexes and histone deacetylases (HDACs), promoting chromatin compaction and transcriptional silencing (27). MBD1 additionally contains three CxxC zinc-finger motifs that confer binding capacity at both methylated and unmethylated CpG sites. This dual DNA-binding capability enables MBD1 to participate in the regulation of both DNA methylation and histone modifications, thereby exerting broader effects on chromatin organization (28). MBD2, by contrast, contains glycine-rich regions that facilitate assembly of the NuRD complex and enhance recognition of methylated CpG sites within this chromatin-remodeling complex (29).

Unlike other MBD family members, MBD4 functions primarily as a thymine DNA glycosylase owing to the presence of a C-terminal glycosylase domain (30). This domain mediates recognition and excision of thymine or uracil mismatches at CpG dinucleotides, thereby initiating base excision repair and contributing to maintenance of genome integrity (27, 31, 32).

MBD3 is functionally distinct from canonical MBD family members, as a critical amino acid substitution within its MBD domain markedly reduces its affinity for methylated CpG DNA (5mC). Consequently, MBD3 does not operate primarily through sequence-specific recognition of methylated DNA. Instead, it functions predominantly as a chromatin-associated regulatory factor within the NuRD complex, where it modulates chromatin accessibility, histone modifications, and transcriptional programs (33). In contrast to MeCP2 and MBD2, which are broadly associated with transcriptional repression, MBD3 exhibits context-dependent regulatory activity and can function as either a transcriptional activator or repressor, depending on cellular states and microenvironmental context (19). These properties distinguish MBD3 from other family members and underscore its role as a key mediator of epigenetic plasticity and chromatin regulation.

Collectively, MBD proteins function as molecular interpreters of DNA methylation signals, recruiting chromatin-modifying complexes that establish context-dependent chromatin states, ranging from transcriptionally repressive heterochromatin to permissive euchromatin (25). Through these activities, the MBD family serves as a critical mechanistic link between DNA methylation and chromatin remodeling, contributing both the stable maintenance and dynamic modulation of gene expression in response to developmental, cellular, and environmental cues.

**Figure 2:**
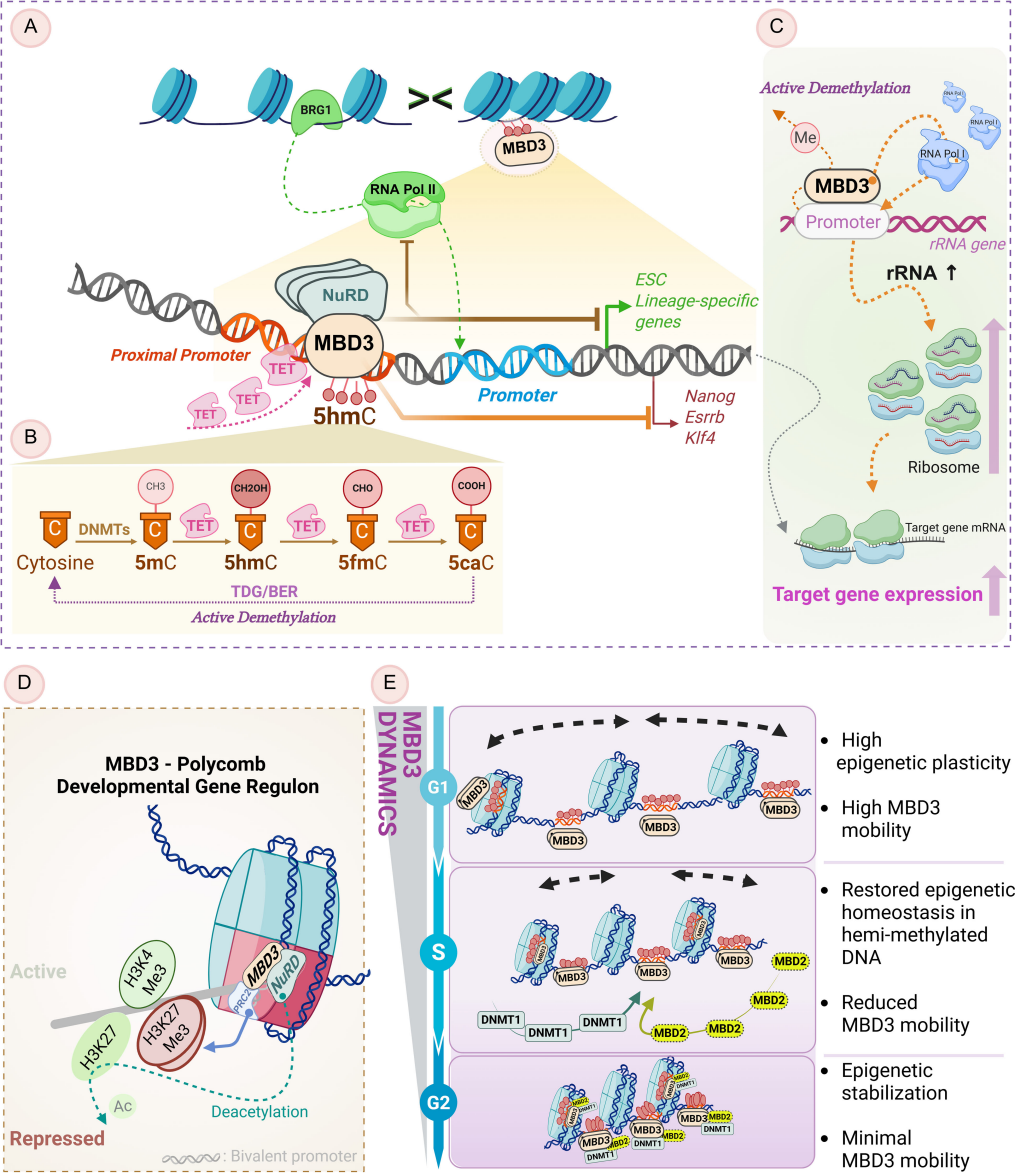
Epigenetic mechanisms mediated by MBD3 and their coordination with chromatin state transitions throughout the cell cycle. **(A)** MBD3/NuRD-dependent regulation of stemness and lineage specification. In decondensed chromatin, MBD3 occupies 5hmC-marked proximal promoters and recruits the NuRD complex to suppress pluripotency-associated transcription factors (including NANOG, ESRRB, KLF4) or to repress lineage specification genes by preventing BRG1-mediated RNA Polymerase II (RNA Pol II) recruitment. These regulatory activities operate in a context-dependent manner and are responsive to environmental cues, enabling dynamic control of stemness maintenance and differentiation programs. **(B)** Cytosine methylation dynamics and TET-mediated active demethylation in healthy cells. DNA methylation at CpG dinucleotides is established when DNA methyltransferases (DNMTs) convert cytosine (C) to 5-methylcytosine (5mC). Active demethylation proceeds through sequential oxidization steps catalyzed by TET family dioxygenases (TET1/2/3), which serially convert 5mC into 5-hydroxymethylcytosine (5hmC), 5-formylcytosine (5fC), and 5-carboxylcytosine (5caC). The terminal oxidized intermediates 5caC is subsequently excised by thymine DNA glycosylase (TDG) and restored to unmodified cytosine through base excision repair (BER). **(C)** MBD3-dependent regulation of ribosomal RNA gene expression and ribosome biogenesis. MBD3 participates in active DNA demethylation at rRNA gene promoters, maintaining a hypomethylated chromatin state permissive for transcription. This is associated with enhanced RNA Polymerase I (RNA Pol I) occupancy at rRNA promoters, increased ribosome biosynthesis, and upregulated expression of target genes. **(D)** Cooperative regulation of developmental gene programs by MBD3/NuRD and Polycomb repressive complexes. MBD3 occupancy at bivalent promoters facilitates PRC2 recruitment and H3K37 trimethylation (H3K27Me3), establishing transcriptionally repressive chromatin states at target loci. Concomitantly, NuRD-mediated histone deacetylation reinforces chromatin compaction and consolidates transcriptional silencing, synergistically coupling MBD3/NuRD and Polycomb-dependent repression at shared genomic targets. **(E)** Cell-cycle–dependent dynamics of MBD3 chromatin occupancy and its coordination with DNMT- and MBD2-mediated epigenetic maintenance during DNA replication. Created with BioRender.com.

Consistent with its chromatin-regulatory functions, MBD3 has been implicated in multiple developmental and cell fate-associated processes, including pluripotency maintenance, cellular reprogramming, and embryonic lineage commitment (24, 34, 36, 37). In the nervous system, MBD3/NuRD regulates the expression of neurogenesis-associated genes involved in neuronal differentiation and maturation, maintaining these loci in a transcriptionally poised state during early developmental stages (21). This regulatory mechanism suppresses premature neuronal differentiation while preserving neural progenitor identity, implicating MBD3 in the coordination of neurodevelopmental timing and stem cell maintenance.

Beyond its preferential association with 5hmC-enriched chromatin, MBD3 also occupies promoters, enhancers, and gene bodies of actively transcribed loci (38). Emerging evidence further suggests that MBD3 participates in the maintenance of DNA methylation homeostasis through functional coordination with DNA methyltransferases (DNMTs) and TET enzymes, particularly within demethylation-prone genomic regions (35) (**Figure 2B**). Consistent with this role, MBD3 associates with unmethylated ribosomal RNA (rRNA) gene promoters and contributes to the maintenance of their active chromatin state, whereas MBD3 depletion leads to promoters hypermethylation (39). Conversely, MBD3 overexpression has been reported to promote demethylation at rRNA promoter, enhance RNA polymerase I recruitment, and augment ribosome biogenesis (**Figure 2C**). At a broader genomic scale, MBD3 overexpression has been linked to global DNA demethylation, particularly at promoters enriched for intermediate CpG density and nuclear factor Y (NF-Y) binding sites (40).

MBD3 also functionally cooperates with Polycomb-associated chromatin regulatory networks (**Figure 2D**). MBD3/NuRD is enriched at Polycomb-bound loci, particularly CpG-rich promoters marked by H3K27me3 and frequently exhibiting bivalent chromatin features (34). Mechanistically, recruitment of MBD3/NuRD to these promoters facilitates subsequent PRC2 binding and H3K27 trimethylation, thereby reinforcing transcriptionally repressive chromatin states (41, 42). In parallel, NuRD-mediated histone deacetylation establishes a chromatin environment permissive for PRC2-dependent H3K27me3 deposition, functionally coupling MBD3/NuRD activity to Polycomb-mediated gene silencing (41, 43, 44). Reciprocal regulatory interactions between MBD3 and Polycomb components further modulate each other’s expression and activity, expanding the epigenetic regulatory capacity of these chromatin-modifying networks (45). Through this physical and functional coordination, MBD3/NuRD and PRC2 cooperatively stabilize repressed or poised transcriptional states during lineage specification and developmental regulation, expanding the regulatory capacity of chromatin-modifying networks.

Emerging evidence further indicates that MBD3 participates in the maintenance of epigenetic states during cell-cycle progression. MBD3 undergoes dynamic redistribution across the cell cycle and coordinates with DNMTs and MBD2 during S and G2 phases to support the faithful propagation of locus-specific epigenetic states following DNA replication (35) (**Figure 2E**). During G1 phase, increased 5mC–5hmC interconversion is associated with elevated chromatin plasticity, enhanced MBD3 mobility, and preferential localization to 5hmC-rich regions. During S phase, DNA replication generates hemi-methylated intermediates in which the nascent strand lacks epigenetic marks. DNMT1-meidated restoration of 5mC on the newly synthesized strand occurs in parallel with differential recruitment of MBD2 and MBD3 to 5mC- and 5hmC-enriched regions, respectively, thereby contributing to the faithful reestablishment of DNA methylation patterns. During G2 phase, MBD3 exhibits more stable chromatin association coincident with progressive chromatin compaction, suggesting a role in consolidating epigenetic architecture prior to mitosis.

**Figure 3:**
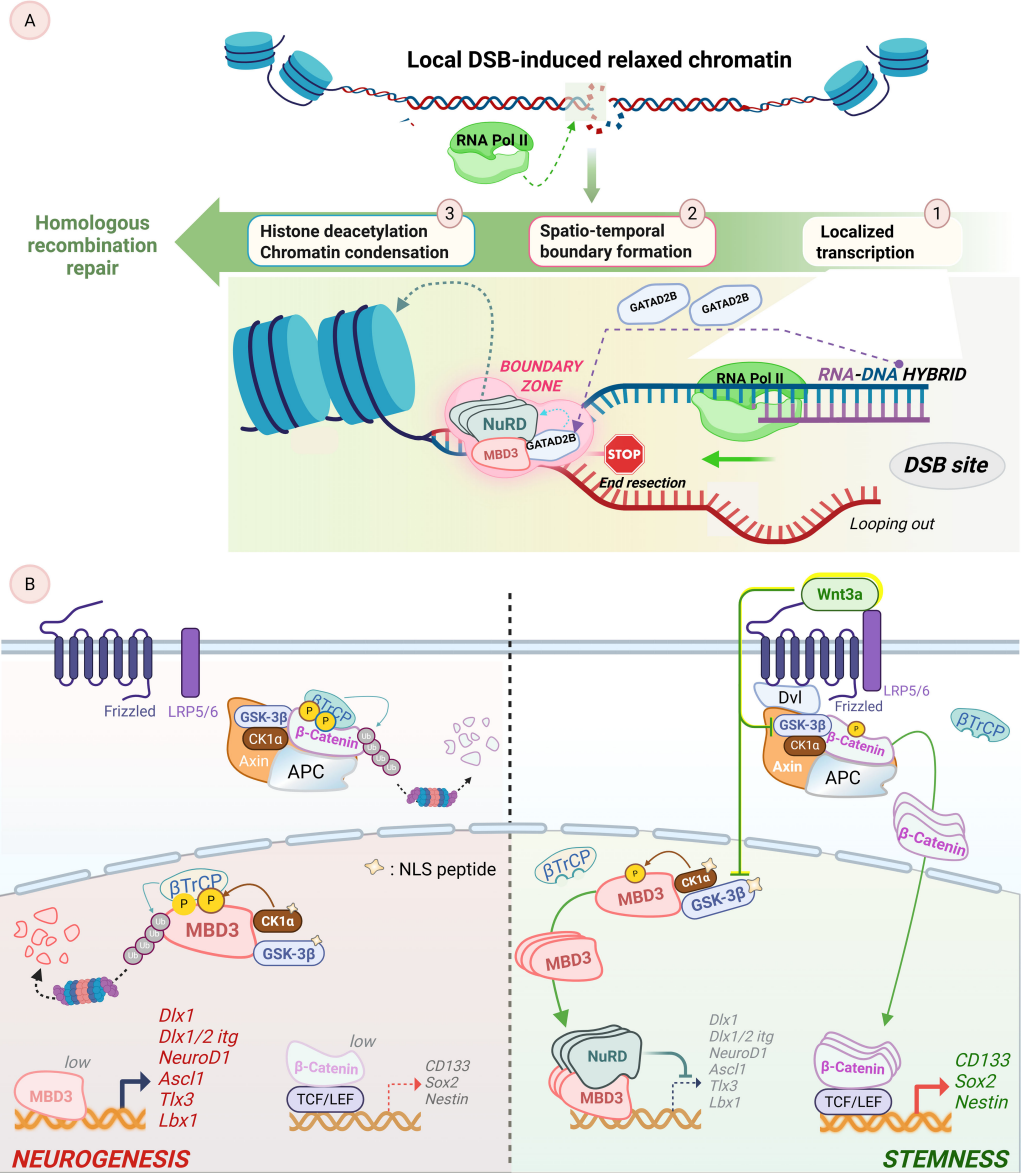
Regulatory roles of MBD3 beyond chromatin remodeling. **(A)** MBD3/NuRD-mediated facilitation of homologous recombination repair at DNA double-strand break (DSB) sites. Following DSB-induced local chromatin relaxation, repair proceeds through three sequential steps (1): RNA-Pol II recruitment to the DSB site initiates localized transcription, generating DNA–RNA hybrids and facilitating GATAD2B recruitment (2); MBD3/NuRD complexes are recruited to GATAD2B-occupied regions, establishing a spatially defined chromatin boundary zone; and (3) NuRD-mediated histone deacetylation and chromatin remodeling organize local chromatin architecture to facilitate homologous recombination-mediated repair. **(B)** Divergent downstream outputs of canonical Wnt signaling mediated by MBD3 and β-catenin during neurogenesis. MBD3 repress neurodifferentiation-associated gene programs, whereas β-catenin drives transcriptional activation of stemness-related genes, collectively fine-tuning neural progenitor cell fate decisions. Created with BioRender.com.

In addition to its chromatin-regulatory functions, MBD3 participates in several broader cellular processes. MBD3 has been implicated in apoptosis regulation in mouse embryonic stem cell (20) and has more recently been identified as a mediator of DNA double-stranded break (DSB) repair (**Figure 3A**). At sites of DNA damage, the MBD3/NuRD complex establishes spatiotemporal chromatin boundaries and promotes histone deacetylation, inducing chromatin condensation that terminates DNA end resection and facilitates efficient homologous recombination-mediated repair (46). MBD3 has additionally emerged as a signaling-responsive regulatory factor in developmental pathways, functioning as a downstream effector of canonical Wnt signaling in parallel with β-catenin (22) (**Figure 3B**). The Wnt–Mbd3 axis augments Wnt pathway output and contributes to neural progenitor cell homeostasis, which is essential for normal neurodevelopment and behavior.

Collectively, these findings underscore the multifaceted involvement of MBD3 in coordinating chromatin remodeling, DNA methylation dynamics, and transcriptional regulation. Through the integration of multiple epigenetic pathways, MBD3 emerges as a critical molecular nexus linking cellular plasticity, lineage specification, and developmental homeostasis across diverse biological contexts.

## 3 MBD3 in GBM heterogeneity and therapeutic resistance

### 3.1 MBD3 expression and epigenetic states in GBM

GBM exhibits profound intra- and inter-tumoral heterogeneity, representing one of the principal barriers to durable therapeutic response. This heterogeneity arises through the interplay of genetic alterations, epigenetic remodeling, lineage plasticity, and microenvironmental adaptation, collectively generating tumor cell populations with distinct proliferative capacities, invasive properties, and therapeutic vulnerabilities. Foundational driver alterations, including mutations in *IDH1/2*, *TERT* promoter, *EGFR*, *PTEN*, and *TP53*, establish divergent subclonal lineages that subsequently undergo further diversification through mismatch repair defects and therapy-induced mutagenesis (47–49). Dynamic interactions between neoplastic cells and surrounding neural, glial, and immune populations further contribute to regional and temporal variability within the tumor ecosystem (50, 51).

A major determinant to this adaptive heterogeneity is cellular plasticity. Neural stem- and progenitor-like populations that acquire oncogenic alterations while retaining self-renewal capacity give rise to glioma stem-like cells (GSCs), which are enriched in DNA repair pathways, stress-response programs and chemoresistance associated signaling networks (52). Under intrinsic and therapy-imposed selective pressure, GBM cells can additionally undergo dynamic state transitions, including proneural-to-mesenchymal switching and epithelial-to-mesenchymal transition (EMT)-like programs, thereby acquiring enhanced migratory, invasive, and therapy-resistant phenotypes. These adaptive transitions frequently overlap with GSCs -associated functional states and are increasingly recognized as major drivers of tumor recurrence.

Given the central role of epigenetic plasticity in governing these processes, increasing attention has focused on chromatin regulators that coordinate subtype-specific transcriptional states in GBM. Among these, MBD3 has emerged as a candidate regulator of glioma heterogeneity. Although current evidence does not establish a definitive association between MBD3 mutational status or expression levels and patient survival, malignant glioma tissues display strong MBD3 immunopositivity relative to other tumor types, suggesting a potential contribution of MBD3 to gliomagenesis (53). Human patient-derived tissue microarray analyses further demonstrated that MBD3 protein abundance increases in a grade-dependent manner and is important for the maintenance of putative GSC populations and tumorigenicity (17, 18). Consistently with these findings, data from the Human Protein Atlas indicate strong nuclear MBD3 immunoreactivity in most high-grade malignant gliomas, whereas lower-grade tumors exhibit more heterogenous expression patterns (53).

An apparently contrasting observation was reported by Cui et al., in which MBD3 chromatin occupancy decreased with increasing glioma grade and was accompanied by reduced enrichment of 5hmC (16) - a major MBD3-associated chromatin mark in the brain (54). Patients exhibiting high intratumoral co-enrichment of MBD3 and 5hmC demonstrated improved progression-free survival and overall survival following gross total resection and adjuvant therapies, whereas reduced co-enrichment was associated with more aggressive, pre-multifocal growth patterns. A survival-proportional contribution of MBD3 and 5hmC was consistently observed in WHO Grade II and III gliomas. Although MBD3 depletion significantly enhanced the migration and proliferation of one glioblastoma cell line *in vitro*, the absence of multi-line validation limits the generalizability of these findings to GBM biology more broadly. The discrepant observations regarding grade-dependent MBD3 levels likely reflect confounding factors, including differences in patient treatment history, intrinsic genetic and epigenetic heterogeneity, tumor cellular composition, antibody isoform specificity, and variability in tumor sampling and TMA quantification criteria. Furthermore, as GSC populations were not specifically examined in this study, these findings collectively suggest that MBD3 function may be governed less by absolute expression levels than by the surrounding epigenetic landscape and tumor cell context.

**Figure 4:**
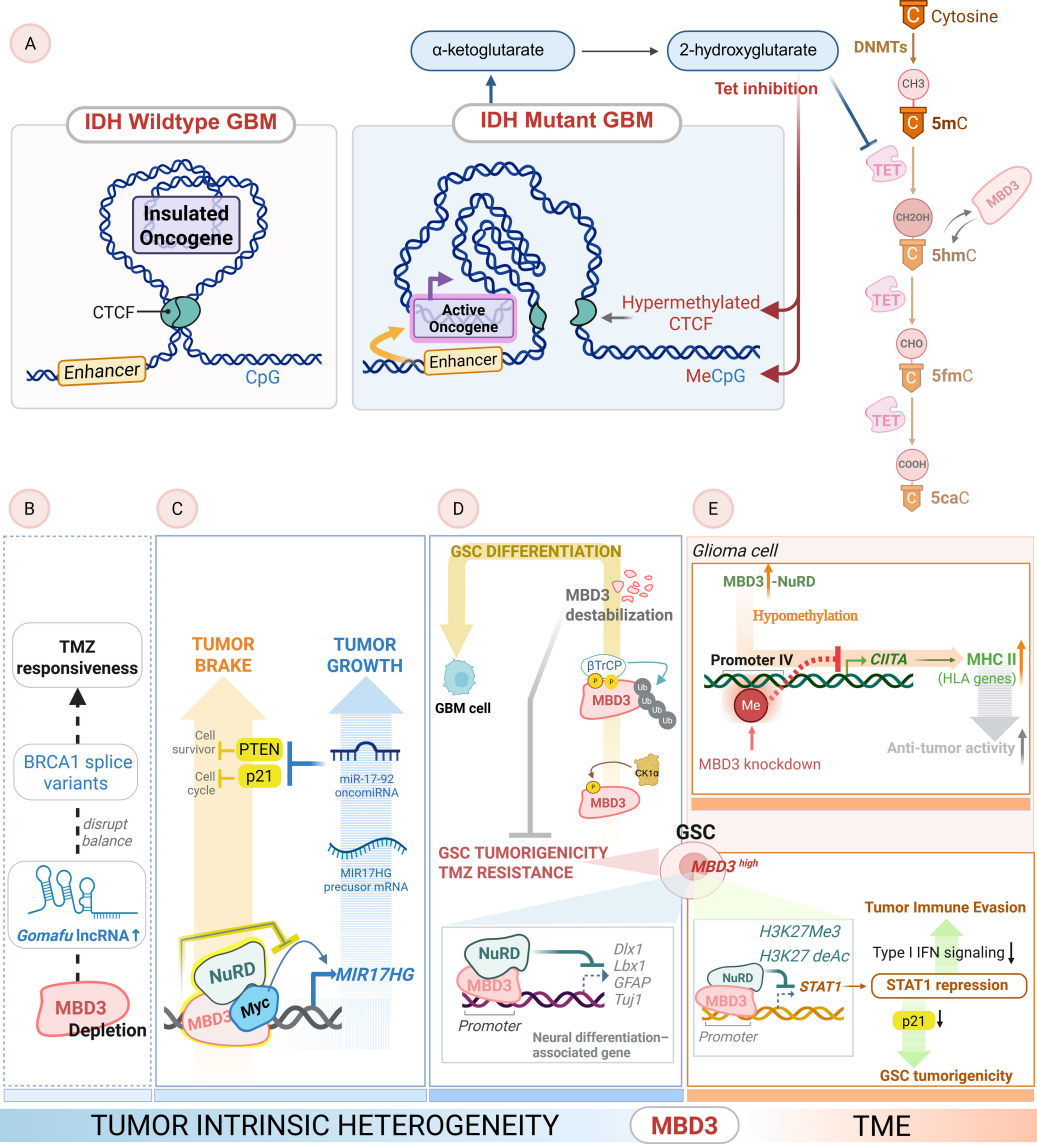
Direct regulatory roles of MBD3 in GBM heterogeneity. **(A)** Proposed association between MBD3 activity and *IDH*-mutant GBM through disruption of 5hmC homeostasis. In *IDH1*-mutant glioma, neomorphic IDH1 activity diverts α-ketoglutarate to the oncometabolite 2-hydroxyglutarate, which competitively inhibits TET dioxygenases and impairs 5mC-to 5hmC conversion (57). Consequent 5mC accumulation drives CpG island hypermethylation and the glioma CpG island methylator phenotype (55). Hypermethylation at CTCF boundary elements disrupts topologically associating domain (TAD) insulation, enabling aberrant enhancer and super-enhancer activity over oncogenes and gliomagenesis-associated gene loci. **(B–D)** MBD3-dependent mechanisms governing tumor intrinsic heterogeneity. **(B)** MBD3 negatively correlates *Gomafu* lncRNA expression, which is hypothetically linked to *BRCA1* alternative splicing and modulation of GBM cell responsiveness to TMZ. **(C)** MBD3/NuRD attenuates MYC/MIR17HG-driven transcriptional activation of the miR-17–92 cluster, suppressing its pro-tumorigenic effects on cell growth and survival and thereby exerting tumor-suppressive activity. **(D)** MBD3 preserves GSC identity and supports GBM progression and therapeutic resistance. CK1α/β-TrCP-dependent polyubiquitination and proteasomal degradation of MBD3 relieves NuRD-mediated repression of neuronal differentiation programs, impairing GSC self-renewal capacity. **(E)** MBD3-dependent modulation of GBM immune heterogeneity through regulation of tumor microenvironment (TME) interactions. (Lower panel) MBD3/NuRD recruitment to the *STAT1* promoter in GSCs suppresses *STAT1* transcription, attenuating type I interferon signaling to promote immune evasion and reducing p21-mediated cell-cycle inhibition to enhance GSC proliferation and tumorigenicity. (Upper panel) In GBM cells, MBD3 occupancy at promoter IV of the *CIITA* gene (class II major histocompatibility complex transactivator gene) maintains a hypomethylated chromatin state, promoting *CIITA* transcription, upregulating *HLA* variant transcripts, and enhancing MHC-II surface expression, a mechanism hypothetically linked to anti-tumor immune recognition. Panel **(B–E)**: Dashed border indicates not fully experimentally validated; solid border indicates experimentally validated results. Created with BioRender.com.

Such context dependency is particularly relevant in GBM, where DNA methylation dynamics are closely linked to molecular subtype, most notably *IDH* mutational status. In *IDH-*mutant gliomas, sustained DNMT-mediated methylation combined with impaired TET-dependent demethylation drives progressive CpG island hypermethylation, ultimately producing the glioma CpG island methylator phenotype (G-CIMP) (55, 56) (**Figure 4A**, right panel). Given that glioblastoma is now a designation reserved exclusively for *IDH*-wildtype grade 4 tumors under the WHO CNS5 classification (**Table 1**), TET inhibition and the consequent global reduction of 5hmC in IDH-mutant disease putatively attenuates MBD3 chromatin engagement in lower-grade astrocytomas rather than in true glioblastoma. This interpretation is consistent with reported associations between MBD3 and GSC maintenance and gliomagenesis, and may partially account for the differential MBD3 chromatin occupancy observed across glioma grades. Crucially, under the updated WHO CNS5 framework, adult diffuse gliomas are now strictly stratified by molecular phenotype (**Table 1** (58)) - a paradigm shift that is essential when interpreting MBD3 function across glioma subtypes. Within the epigenetically unstable landscape of true IDH-wildtype glioblastoma, MBD3-dependent regulation of cellular state transitions and microenvironmental immune evasion may be particularly prominent. Importantly, these epigenetic alterations extend beyond generalized transcriptional repression. Flavahan et al. demonstrated that hypermethylation at CTCF boundary elements disrupts topologically associating domain (TAD) insulation, enabling aberrant enhancer-oncogene interactions and constitutive activation of oncogenic transcriptional programs (59) (**Figure 4A**). Complementary studies by Glowacka et al. further identified enrichment of 5hmC at enhancers and super-enhancers associated with oncogenes and gliomagenesis-related transcriptional programs (60, 61). Although certain oncogenic enhancer programs are shared between *IDH*-wildtype and *IDH*-mutant gliomas, distinct 5hmC signatures at these regulatory elements differentiate the epigenetic landscapes of the two subtypes, highlighting the contribution of enhancer-associated epigenetic remodeling to GBM transcriptional diversity.

**Table 1:** Classification of IDH-related gliomas in accordance with World Health Organization classification of tumors of the central nervous system, 5th edition (2021) (WHO CNS5).

Official WHO CNS5		Grade 4		Grade 2/3	
		Glioblastoma, IDH-wildtype	Astrocytoma, IDH-mutant	Astrocytoma, IDH-mutant	Oligodendroglioma, IDH-mutant and 1p/19q-codeleted
Essential Molecular Markers	Definitional Markers	• *IDH1/2* wildtype	• *IDH1/2* mutant	• *IDH1/2* mutant	• *IDH1/2* mutant • *1p/19q* co-deleted
	Molecular Grading Markers	AND ≥1 of the following: • *TERT* promoter mutant • *EGFR* amplified • Combined Chromosome 7 gain/10 loss (+7/-10)	AND ≥1 of the following: • *CDKN2A/B* homozygous deleted • Histological necrosis/MVP	LACK Grade 4 features: • *CDKN2A/B* intact • No necrosis/MVP	• Graded histologically: • No definitive isolated molecular grading marker exists yet.
	Lineage & Supporting Markers	• Histological high-grade features are highly characteristic but not required if molecular markers are positive.	AND all of the following: • ATRX-deficient (nuclear) • *TP53* mutant	AND all of the following: • ATRX-deficient (nuclear) • *TP53* mutant	AND all of the following: • ATRX-retained • *TP53* wildtype • *TERT* promoter mutant
Histological Grading/Phenotypes		• Microvascular proliferation(MVP) • Pseudopalisading necrosis	• Marked nuclear atypia, microvascular proliferation, and/or geographical tissue necrosis.	• Grade 3: High mitotic activity and anaplasia. • Grade 2: Well-differentiated, cellular hyperchromasia but very low mitotic counts.	• Uniform round cells with perinuclear halos (‘fried eggs’) and branching capillaries (‘chicken wire’). • Grade 3: Significant histological anaplasia. • Grade 2: Well-differentiated cells with low mitotic activity.
Pre-2021 Classification		• Primary Glioblastoma Multiforme • Anaplastic Astrocytoma (Grade 3)	• Secondary Glioblastoma Multiforme • Anaplastic Astrocytoma (Grade 3)	• Diffuse Astrocytoma (Grade 2) • Anaplastic Astrocytoma (Grade 3)	• Oligodendroglioma (Grade 2) • Anaplastic Oligodendroglioma (Grade 3)

Given the preferential association of MBD3 with 5hmC-enriched chromatin, these subtype-specific methylation landscapes may partially account for the heterogeneous distribution and context-dependent activity of MBD3 across GBM states. Notably, elevated 5hmC abundance has been associated with increased *LGR5* expression (61), a regulator implicated in cancer stem-like phenotypes (62–64), further linking enhancer-associated hydroxymethylation to stemness-related transcriptional programs. Together, these observations implicate MBD3 in epigenetic regulatory networks governing enhancer activity, lineage plasticity, and subtype-specific transcriptional states in GBM.

Collectively, current evidence supports a model in which GBM heterogeneity is shaped not only by genetic instability but also by dynamic epigenetic remodeling driven by subtype-specific enhancer regulation, chromatin topology, and therapeutic adaptation. Within this framework, MBD3 may function as a context-dependent chromatin regulator whose activity is determined by cellular lineage, molecular subtype, and epigenetic state. The discrepancies observed between MBD3/5hmC enrichment and glioma grade likely reflect differences in subtype composition and temporal reprogramming during tumor evolution, underscoring the need for integrative, subtype-resolved analyses to precisely define the role of MBD3 in GBM progression.

### 3.2 MBD3-dependent regulatory programs in GBM cells

Current findings position MBD3 not merely as a passive chromatin-associated factor, but as an active modulator of adaptive tumor cell states, regulating diverse aspects of intrinsic proliferative and fate decision within GBM. Accumulating evidence supports a central role for MBD3 in maintaining putative GSC properties and stemness-associated transcriptional programs, as well as driving malignant progression (17, 18), collectively implicating MBD3 as an integral component of the GBM epigenetic regulatory network.

One of the earliest mechanistic links between MBD3 and GBM regulatory circuitry was described by Cui et al., who demonstrated that MBD3 negatively regulates the nuclear long non-coding RNA (lncRNA) *Gomafu* (*MIAT*), a modulator of alternative splicing (16) (**Figure 4B**). MBD3 depletion resulted in substantial upregulation of *Gomafu*, which was associated with altered splicing of tumor-suppressor gene *BRCA1*, a major determinant of temozolomide (TMZ) responsiveness. The author proposed that disruption of the stoichiometric balance among BRCA1 splice variants following MBD3 loss may contribute to altered therapeutic sensitivity. However, the proposed MBD3-*Gomafu*-*BRCA1* regulatory axis remains largely correlative, as direct mechanistic causality has not been fully established. Given that MBD3 interacts extensively with promoters and gene bodies to modulate transcription, the observed splicing alterations may reflect broader epigenetic dysregulation consequent to MBD3 loss, rather than arising exclusively through a discrete lncRNA-mediated pathway.

In the same study, MBD3 was further implicated in promoting GBM proliferation through transcriptional activation of the oncogenic miR-17–92 cluster (16). This polycistronic miRNA cluster encodes six mature miRNAs with broad regulatory activities, collectively targeting key regulators of cell-cycle progression, apoptosis, and survival signaling, including *CDKN1A (p21)* and *PTEN*, thereby attenuating proliferative restraint and tumor suppression (**Figure 4C**). In this context, MBD3-NuRD-mediated suppression on the miR-17–92 cluster operates through inhibition of MYC-driven transcription, thereby relieving suppression of PTEN and p21 and reinforcing tumor-suppressive programs. This regulation of oncogenic miRNA networks further implicates MBD3 as a determinant of tumor cell state plasticity in GBM.

Additional mechanistic insight into MBD3-dependent regulation of GSC identity was provided by Moon et al. (18), who identified a CK1α/β-TrCP–dependent ubiquitin–proteasome pathway that destabilizes MBD3 through polyubiquitination. Activation of this signaling axis promotes MBD3 degradation, thereby relieving MBD3/NuRD-mediated transcriptional repression on neurogenesis-associated genes. The resulting induction of neurodifferentiation program compromises GSC stemness and disrupts the self-renewing equilibrium required to sustain tumor-initiating capacity (**Figure 4D**). These findings collectively indicate that tight post-translational regulation of MBD3 abundance is critical for preserving stem-like tumor states in GBM.

Collectively, current evidence indicates that MBD3 functions as a context-dependent regulator of GBM cellular plasticity rather than a strictly unidirectional transcriptional activator or repressor. Through coordinated regulation of non-coding RNA networks, differentiation-associated transcriptional programs, and stemness-related signaling pathways, MBD3 contributes to the maintenance of adaptive tumor cell states and intratumoral heterogeneity. These regulatory functions place MBD3 at the intersection of epigenetic plasticity, therapy resistance, and GSC maintenance, highlighting its potential significance as a driver of GBM progression and recurrence.

## 4 MBD3 in the tumor-extrinsic regulation of GBM

### 4.1 Direct immunoregulatory functions of MBD3 in GBM

The remarkable heterogeneity of GBM arises not only from intrinsic diversity within putative GSC populations but also from dynamic reciprocal interactions between tumor cells and the TME. Through this crosstalk, tumor cells remodel surrounding stromal, immune, and vascular compartments, while the TME simultaneously imposes selective pressures that reinforce lineage specification, invasion, metabolic adaptation, and therapeutic resistance (65). These interactions collectively sustain tumor evolution and contribute substantially to recurrence.

Although direct evidence linking MBD3 to canonical TME-associated processes such as extracellular matrix (ECM) remodeling, angiogenesis and invasion remains relatively limited, accumulating evidence increasingly implicates MBD3 in immune regulatory programs within the GBM microenvironment. In this context, MBD3 may function as an epigenetic regulator that shapes tumor-immune interactions and facilitates immune escape.

Zhan et al. demonstrated that GSCs recruit the MBD3–NuRD complex to repress *STAT1*, thereby attenuating type I interferon signaling and suppressing interferon-stimulated transcriptional programs (17) (**Figure 4E**, lower panel). This epigenetic repression conferred increased resistance to interferon (IFN)-mediated growth suppression, collectively promoting tumor initiation and immune evasion. MBD3-NuRD-mediated STAT1 silencing further resulted in downregulation of the cell cycle inhibitor p21, thereby reinforcing GSC maintenance and GSC-driven tumorigenesis. These findings indicate that MBD3-dependent chromatin remodeling establishes an immunosuppressive transcriptional state that protects GSC populations from immune surveillance.

Complementary evidence suggests that MBD3 may also influence antigen presentation pathways. Cui et al. reported that MBD3/NuRD promotes MHC-II expression in GBM cells by maintaining a hypomethylation state at promoter IV of the MHC-II transactivator *CIITA* gene (16) (**Figure 4E**, upper panel). MBD3 knockdown resulted in promoter IV methylation, leading to transcriptional suppression of *HLA* variants and consequently reduction in MHC-II molecules. The author linked MBD3 depletion-associated MHC-II downregulation to immune evasion during gliomagenesis, supported by significantly lower transcription of *HLA* (MHC II) components in high-grade relative to low-grade gliomas. Although the epigenetic mechanism underlying MBD3-dependent *CIITA* regulation was established, whether this axis functionally governs anti-tumor immune activity requires further validation, as the tumor size experiments employed T-cell deficient mice, which precludes assessment of MHC-II-mediated antigen presentation in an intact immune context. Together, these observations suggest that MBD3 contributes to immune remodeling in GBM through coordinated suppression of interferon-responsive programs and context-dependent modulation of antigen presentation machinery.

### 4.2 MBD3-associated glioma stem cell-microenvironment crosstalk

Beyond its direct immunoregulatory effects, MBD3 may additionally influence the GBM microenvironment indirectly through maintenance of GSC-associated transcriptional programs. Elevated MBD3 expression has been associated with stem-like GBM populations and high-grade gliomas, including enrichment in triple-positive CD44^+^CD133^+^CXCR4^+^ GSC populations (18). CD44, CD133, and CXCR4 are widely recognized as canonical markers of GSC maintenance and tumor aggressiveness (66–70). Importantly, these surface regulators actively reshape TME interactions through diverse downstream signaling cascades, rather than serving merely as phenotypic identifiers. MBD3-dependent maintenance of GSC transcriptional states may therefore indirectly influence TME remodeling. Several signaling pathways associated with GSC maintenance intersect with established MBD3 regulatory networks. Notably, canonical Wnt/β-catenin signaling, frequently activated in stem-like GBM populations, stabilizes MBD3 and promotes its function as a downstream chromatin-associated effector (22). This reciprocal relationship raises the possibility that MBD3 participates in broader transcriptional circuits coupling stemness maintenance with microenvironmental adaptation, positioning it within an MBD3-centered regulatory axis linking GSC identity to TME remodeling.

**Figure 5:**
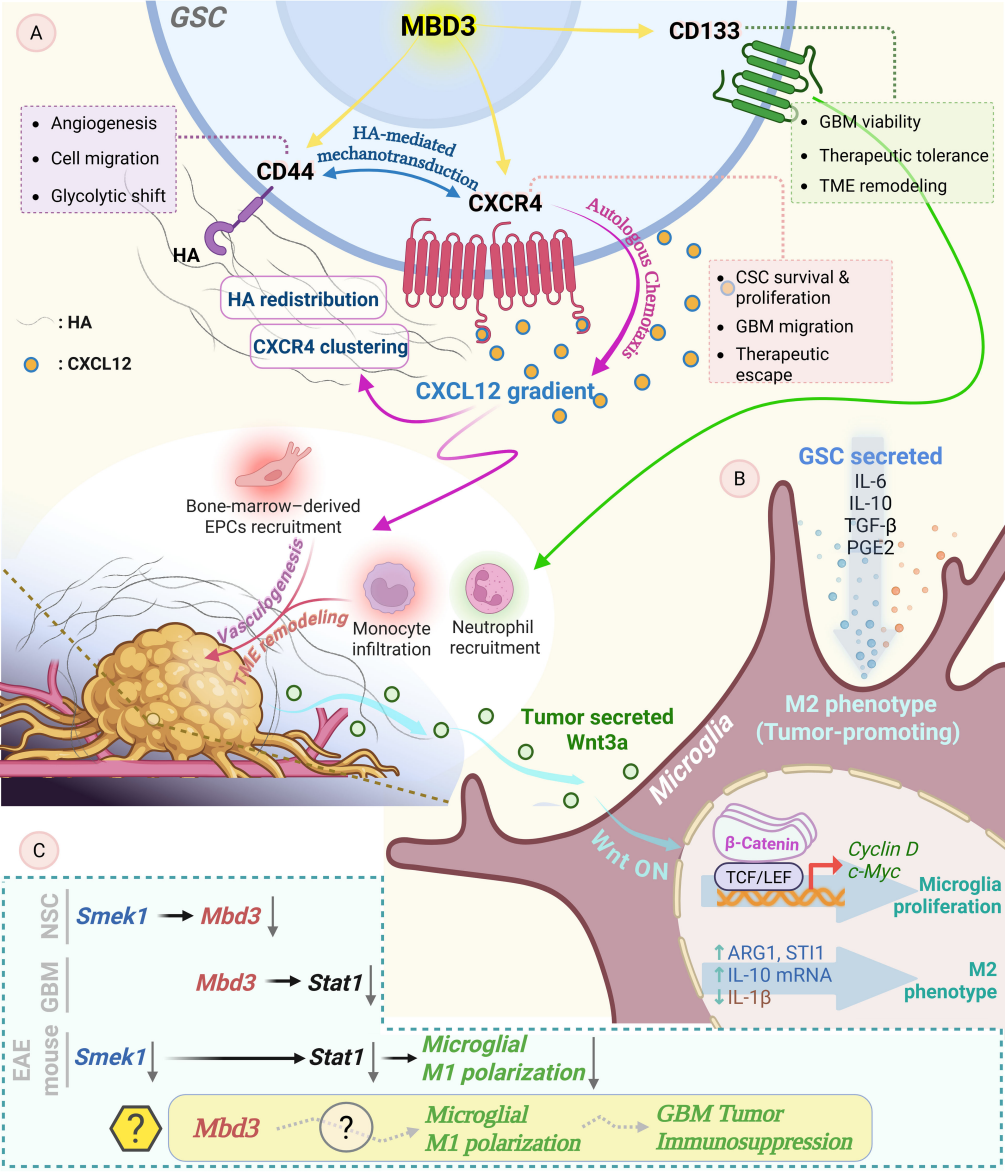
MBD3-centered regulatory network linking GSC-associated programs to TME remodeling and microglial modulation. **(A)** MBD3-dependent maintenance of GSC marker expression — encompassing CD44, CXCR4, and CD133 — and their downstream signaling cascades governing TME remodeling. CD44–hyaluronan (HA) interactions promote tumor invasion and therapeutic resistance; CXCR4–CXCL12 signaling drives directional migration, vasculogenesis, and immune evasion; CD133-associated pathways sustain stemness, survival, and immunomodulation. **(B)** GSC- and GBM cell-driven polarization of tumor-associated microglia within the TME, mediated through Wnt3a/β-catenin signaling and GSC-derived immunosuppressive cytokines, collectively promoting pro-tumorigenic microglial states. **(C)** Flow charts summarizing experimentally validated MBD3-associated signaling pathways across distinct neural systems (NSC, GBM, EAE mouse). The yellow box presents a hypothetical mechanistic framework for MBD3-mediated modulation of microglial polarization, integrating MBD3/NuRD-dependent STAT1 repression with Wnt/β-catenin-driven transcriptional reprogramming. Created with BioRender.com.

Among GSC-associated surface regulators, CD44 represents a principal mediator of tumor-matrix interactions and adaptive niche remodeling (**Figure 5A**, left) (18, 68). CD44 interacts extensively with hyaluronan (HA), a major ECM component enriched within the GBM microenvironment (71). Activation of the CD44-HA axis promotes migration, invasion, and resistance to both chemotherapy and radiotherapy (72–74). This signaling network additionally contributes to metabolic reprogramming by enhancing glycolytic adaptation and attenuating oxidative stress, thereby supporting tumor survival within hypoxic regions of the GBM core (74). Through these coordinated effects, CD44 signaling reinforces metabolically flexible and invasive GSC phenotypes capable of adapting to hostile microenvironmental conditions.

CXCR4 similarly exerts broad regulatory effects on GSC behavior and TME remodeling (**Figure 5A**, middle). As the canonical receptor for CXCL12 (SDF-1), CXCR4 signaling supports stem-like maintenance, proliferation, and survival through activation of PI3K–AKT signaling, anti-apoptotic programs, and cell-cycle regulators including Cyclin D1, CDK4/6, particularly under hypoxic stress (75, 76). GBM cell migration (77, 78) and therapeutic escape (79) have similarly been linked to CXCR4 signaling activity. Beyond these cell-intrinsic functions, CXCR4 shapes the surrounding microenvironment by promoting vasculogenesis and recruitment of circulating bone marrow-derived endothelial progenitors following radiotherapy, thereby facilitating post-treatment tumor recurrence (80). Pharmacological inhibition of the CXCR4-CXCL12 axis suppresses vascular regeneration and inflammatory cells recruitment, markedly impairing tumor regrowth in both cell line-derived and xenograft models (76). Furthermore, CXCR4 signaling contributes to proneural-to-mesenchymal transition (PMT), a plasticity-associated process closely linked to invasion and therapeutic resistance (81).

An additional layer of complexity is introduced by interstitial fluid flow (IFF), a biomechanical feature of the GBM microenvironment that governs long-range cellular communication and directional migration. IFF enhances GSC invasiveness through coordinated autologous CXCR4-CXCL12-mediated chemotaxis and HA-mediated mechanotransduction via CXCR4-CD44 axis (82). Locally generated CXCL12 gradients within the tumor induce ECM deformation and pericellular HA redistribution, producing membrane tension changes that spatially reorganize CXCR4 at the leading edge of migrating cells. This receptor clustering amplifies local sensitivity to CXCL12 and activates downstream PI3K–AKT and MAPK/ERK signaling, promoting cytoskeletal remodeling, directional migration, and sustained stem-like transcriptional programs. These observations illustrate how biochemical and biomechanical cues are integrated to drive adaptive tumor cell behavior within the GBM microenvironment.

CD133 further contributes to this interconnected regulatory landscape (**Figure 5A**, right). Although widely employed as a neural stem cell and GSC marker, CD133 also functions upstream of multiple oncogenic signaling cascades that collectively reinforce stemness, survival, and recurrence, including Wnt/β-catenin, telomerase/TERT and AKT signaling, thereby promoting treatment resistance, and sustaining long-term tumor propagation (83–86). CD133-positive populations display remarkable resilience under diverse microenvironmental stresses, including hypoxia and therapeutic challenge. These cells preferentially expand under hypoxic conditions (87), evade rapamycin-induced apoptosis through autophagy activation (88), and initiate TMZ resistance even under normoxic conditions (89). In addition, CD133-positive GSCs may exert immunomodulatory effects through IL-1β–mediated recruitment of neutrophils and other inflammatory cells, further reshaping the local immune environment (90).

Collectively, these findings suggest that MBD3-dependent maintenance of GSC-associated transcriptional states may indirectly orchestrate broad microenvironmental remodeling in GBM. Through coordinated regulation of CD44, CXCR4, and CD133- associated stemness programs, MBD3 may contribute to the establishment of invasive, therapy-resistant, and metabolically adaptable tumor niches. Notably, the coupled CD44–CXCR4 signaling network exemplifies how GSCs integrate biochemical and biomechanical cues to remodel their microenvironment, linking stemness maintenance with invasion, plasticity, and adaptive tumor behavior. Together, these interactions position MBD3 at the intersection of epigenetic plasticity, GSC maintenance, and TME adaptation, reinforcing its potential significance as a driver of GBM progression and recurrence.

## 5 Discussion

### 5.1 Microglial plasticity as a potential context for MBD3-dependent immune remodeling

The capacity of GBM cells to hijack and remodel the TME is mediated through a highly intricate network of cellular and molecular interactions, among which immune components play central roles in establishing immune evasion and driving tumor recurrence. Although the majority of immune cells recruited to GBM tumor mass originate from peripheral infiltration, microglia - the specialized resident immune sentinels of the CNS - constitute the predominant population of mononuclear phagocytes within the tumor and persist throughout the entire course of glioma development, from initiation to progression (91). Microglia are therefore uniquely positioned to exert sustained influence on tumor evolution and immune adaptation within the CNS microenvironment.

Microglia are frequently grouped with infiltrating macrophages under the collective designation of tumor-associated microglia/macrophages (TAMs), as both populations exhibit substantial functional plasticity and can acquire either pro-inflammatory or immunosuppressive phenotypes (classically characterized as M1-like antitumor versus M2-like immunosuppressive states). However, accumulating evidence indicates that microglia and bone marrow-derived macrophages are not functionally interchangeable within GBM. Dumas et al. demonstrated that conditioned media derived from primary patient‐derived GBM cultures selectively activated mTOR signaling in microglia but not in infiltrating macrophages (92). This microglia-specific response subsequently triggered an mTOR–STAT3/NFκB immunosuppressive cascade that promoted profound T-cell suppression and facilitated immune escape. These findings indicate that resident microglia possess distinct signaling sensitivities that enable tumor cells to establish specialized immunosuppressive interactions within the CNS.

Beyond immune modulation, microglia additionally fulfill non-redundant functions that directly support tumor progression. Resident microglia, but not infiltrating macrophage, provide critical pro-angiogenic signals that facilitate neovascularization within the glioma microenvironment, thereby sustaining tumor expansion (93). Emerging evidence from brain metastasis models further highlights the unique context-dependent plasticity of microglia in response to malignant cues. In breast cancer brain metastasis model, Pukrop et al. demonstrated that microglia can function both as active transport facilitators and migratory guides for tumor cells invading the brain parenchyma through mechanisms dependent on JNK and noncanonical Wnt signaling (94). Notably, these pro-invasive microglial populations displayed marked morphological and behavioral alterations without acquiring canonical M2-like phenotypes, suggesting that tumor-associated microglial reprogramming extends beyond the conventional M1/M2 polarization framework.

Collectively, these observations underscore that microglia possess specialized and highly plastic functions within brain tumors that cannot be inferred from peripheral macrophage biology. Their long-term CNS residency, distinct signaling responsiveness, and context-dependent functional adaptability position microglia as critical regulators of GBM progression and immune remodeling. These properties raise the further possibility that epigenetic regulators governing cellular plasticity, including MBD3-associated chromatin regulatory networks, may contribute to shaping microglial state transitions within the GBM microenvironment.

### 5.2 Current evidence and unresolved questions regarding MBD3 in microglial regulation

The growing recognition of microglia as indispensable regulators of GBM progression has intensified interest in the transcriptional and epigenetic mechanisms governing their lineage identity, functional plasticity, and tumor-associated reprogramming. Extensive studies have established that transcriptional remodeling plays crucial roles in regulating microglial homeostasis (95), neurodegenerative responses (96), and innate immune activation (97, 98). By contrast, the epigenetic regulators that specifically govern microglial state transitions within the GBM microenvironment remain insufficiently defined.

Current evidence regarding MBD3 in microglia remains limited and, in several respects, inconclusive. Available datasets generally indicate that MBD3 protein is either minimally expressed or undetectable in microglia under both physiological and pathological conditions (16, 99). Human transcriptomic analyses have reported heterogenous MBD3 mRNA abundance across CNS regions enriched for microglial populations (100, 101), yet corresponding protein expression has remained consistently difficult to be detected. Similarly, studies in rat microglia identified detectable *Mbd3* transcripts without clear evidence of protein accumulation (99). A recent comparative proteomic analysis of microglia derived from distinct biological sources likewise detected MBD3 transcripts but did not identify MBD3 as a differentially expressed protein (102). These findings reveal a notable disconnect between transcript abundance and detectable protein expression in microglial populations.

Several mechanisms may account for this discrepancy, including post-transcriptional regulation, reduced translational efficiency, rapid proteasomal degradation, or inherently low protein stability within microglia. Nevertheless, the apparent scarcity of detectable MBD3 protein should not be interpreted as definitive evidence of functional absence. Microglia exhibit profound context-dependent plasticity and undergo substantial transcriptional and epigenetic remodeling in response to inflammatory, metabolic, or tumor-derived signals. It therefore remains plausible that MBD3 expression may emerge transiently under specific activation states or within specialized microglial subpopulations that have not yet been adequately characterized.

The recurrent detection of MBD3 transcripts in the absence of robust protein expression additionally raises the possibility of non-canonical regulatory functions at the RNA level. One speculative possibility is the existence of circular or non-coding MBD3-derived RNA variants that may participate in transcriptional or post-transcriptional regulatory networks independently of canonical MBD3 protein function. Although such mechanisms remain entirely hypothetical, these observations collectively underscore the substantial gaps that persist in our understanding of whether MBD3 contributes directly, indirectly, or conditionally to microglial regulation within the GBM microenvironment.

### 5.3 Hypothetical models linking MBD3, GSCs and microglial remodelling

MBD3 may influence how tumor cells reprogram surrounding microglia and establish immunosuppressive microenvironmental states by shaping the transcriptional identity, secretory landscape, and immunomodulatory programs of GSCs. One pathway through which such interactions may operate involves canonical Wnt/β-catenin signaling (**Figure 5B**). Matias et al. demonstrated that both recombinant and tumor-derived Wnt3a promotes nuclear β-catenin accumulation in microglia, inducing Wnt-responsive target genes including *Cyclin-D1* and *c-Myc*, thereby enhancing microglial proliferation (103). Canonical Wnt activation additionally promoted acquisition of tumor-supportive phenotypes through induction of ARG1, STI1 proteins, and IL-10 mRNA while suppressing IL-1β expression. Beyond transcriptional reprograming, β-catenin signaling enhanced membrane nanotube formation in microglia, facilitating tumor-directed intercellular communication, and ultimately accelerating glioma progression in xenograft models. These findings indicate that GBM-derived Wnt ligands can actively remodel microglial behavior toward pro-tumorigenic states.

In parallel, GSCs secrete multiple immunomodulatory mediators, including IL-6, IL-10, TGF-β, and prostaglandin E2 (PGE2), which collectively promote immunosuppressive, M2-like microglial polarization (104, 105). A central downstream mediator of these pathways is indoleamine 2, 3-dioxygenase 1 (IDO1), whose expression is induced by inflammatory and immunosuppressive cytokine signaling and is strongly associated with the establishment of immune tolerance within the TME (106). These observations raise the possibility that MBD3-dependent transcriptional regulation in GSCs may indirectly shape microglial phenotypes by modulating cytokine secretion and downstream immunoregulatory pathways.

Further mechanistic convergence may involve interactions among MBD3, STAT1 signaling and IDO1-associated immune regulation (**Figure 5C**). Duan et al. demonstrated that SMEK1 deficiency impairs IFN-γ/STAT1 signaling by reducing STAT1 phosphorylation, thereby suppressing *STAT1*-dependent transcriptional activation of *IDO1* and consequently attenuating IDO1 enzymatic activity (107). Downstream of this cascade, reduced kynurenine-AHR signaling diminished AHR nuclear translocation and decreased IL-10 transcription, ultimately shifting microglia toward a pro-inflammatory M1-like phenotype. Intriguingly, the MBD3-NuRD complex has previously been shown to repress *STAT1* transcription through promoter occupancy (17), while SMEK1 has been reported to stabilize MBD3 protein (21). These observations collectively suggest the existence of a putative SMEK1–MBD3–STAT1 regulatory axis that may influence microglial polarization within the GBM microenvironment.

Within this framework, at least two non-mutually exclusive models can be envisioned. In the first, MBD3-mediated repression of *STAT1* may attenuate canonical IFN-γ-driven inflammatory programs, thereby limiting M1-like microglial activation and favoring tumor-supportive immune states. In the second, *STAT1* suppression may engage immunoregulatory targets such as *IDO1*, altering kynurenine–AHR–IL-10 signaling and driving broader immunoregulatory remodeling of the TME. The relative contributions of these STAT1-associated pathways in GBM remains unresolved and warrants systematic investigation.

An additional unresolved question concerns whether GBM-derived canonical Wnt signaling could stabilize MBD3 within microglia, analogous to Wnt-dependent MBD3 stabilization reported in other cellular contexts. If such regulation occurs, transient or context-specific induction of MBD3 in tumor-associated microglia may represent an additional layer of epigenetic plasticity contributing to immune adaptation within GBM. Collectively, these hypothetical models position MBD3 as a potential upstream coordinator linking GSC-associated transcriptional programs to microglial reprogramming and immune remodeling in the glioma microenvironment.

### 5.4 Current limitations and future perspectives

Conflicting reports regarding the association between MBD3 abundance and glioma grade likely reflect regional heterogeneity, subtype-specific cellular composition, and differences in the genetic and epigenetic landscapes of GBM. These inconsistencies underscore the need for subtype-resolved analyses that integrate spatial and temporal tumor contexts. Advanced methodological approaches, including single-cell and spatial multi-omics profiling, alongside ATAC-seq and CUT&RUN, may help delineate MBD3-dependent chromatin regulatory programs across distinct tumor cell states and microenvironmental niches.

Current evidence supports MBD3 as a potentially tractable epigenetic target in GBM. However, its broad roles in normal development and chromatin regulation raise legitimate concerns regarding systemic toxicity and off-target effects. The context-dependent nature of MBD3 activity may nonetheless provide opportunities for pathway-selective or temporally controlled therapeutic intervention, emphasizing the importance of dynamic, cell-state–resolved models of GBM biology for rational therapeutic design.

An additional unresolved question is whether MBD3 directly contributes to microglial reprogramming within the GBM microenvironment. If so, such regulation could integrate inflammatory signaling (IFN-γ–STAT1 axis) with metabolic circuitry (IDO1–kynurenine–AhR axis), and MBD3/NuRD-mediated epigenetic control, collectively converging on coordinated immunosuppressive networks. The reported regulation of lncRNAs and miRNAs by MBD3 further suggests that it may operate at the intersection of transcriptional and post-transcriptional programs governing tumor plasticity and tumor–immune crosstalk. Resolving the predominant molecular form of MBD3 in microglia, whether as a stable protein, rapidly degraded peptide, or non-canonical regulatory transcript, will be an important step toward clarifying its potential contribution to tumor-associated microglial states and GBM immune remodeling more broadly.
